# Child participation and care experience in child health clinic nursing encounters: children's views by multi-methodology

**DOI:** 10.1080/17482631.2026.2697603

**Published:** 2026-07-31

**Authors:** Laura Ortju, Ari Haaranen, Päivi Kankkunen, Liisa Karlsson

**Affiliations:** a Department of Nursing Science, University of Eastern Finland, Kuopio, Finland; b Faculty of Educational Sciences, University of Helsinki, Helsinki, Finland

**Keywords:** Care experience, child health clinic, creative methods, multi-methodology, participation, primary health care, study of children's perspectives, role-play, stimulated recall interview, storycrafting method

## Abstract

**Purpose:**

The aim of this qualitative descriptive study was to explore young children's perspectives on participation and care experiences in child health clinic nursing encounters. Knowledge of children's experiences and views can help professionals support children's right to participation.

**Methods:**

Data were generated using a multi-method approach that included researcher-initiated role-play, stimulated recall interviews, Storycrafting, painting and drawing. Eleven children aged 5–7 years and 12 parents participated. Children were involved in selecting methods and settings. Thematic and semiotic analyzes were conducted, supported by researcher triangulation. The Consolidated Criteria for Reporting Qualitative Research checklist guided reporting.

**Results:**

Clinic visits emerged as shared and meaningful experiences for children. Children's narratives highlighted their wish to act as active agents, the importance of play, toys and rewards, the need for psychosocial support and proximity to a support person, and the significance of the clinic's physical appearance. Vaccinations, physical measurements and adults' restrictive actions were described as memorable aspects that shaped children's care experiences.

**Conclusions:**

Clinic visits emerged as shared and meaningful experiences for children. Children's narratives highlighted their wish to act as active agents, the importance of play, the need for psychosocial support, and the significance of the clinic's environment. Vaccinations, physical measurements and adults' restrictive actions were described as memorable aspects that shaped children's care experiences.

## Introduction

Child health clinics (CHCs) form a central component of the Finnish primary healthcare system. They provide universal, free-of-charge services and reach nearly all young children and their families. For children aged 5–7 years, CHC visits typically include assessment of growth, development, vision, hearing, psychosocial well-being, family circumstances and vaccination status. These visits therefore involve both routine measurements and interactional situations in which the child may be asked questions, examined, instructed, comforted or supported by parents and professionals.

In addition, a comprehensive assessment of the health and well-being of the entire family is offered by CHCs, with a strong emphasis on prevention. Public health nurses (PHNs), who in Finland are registered nurses with a public health nursing qualification, carry out most of the clinical work, supported by physicians, psychologists and other specialists. Regular health visits (11 within the first two years and annually thereafter until school age) help in finding health problems early, assure continuous care and support the overall well-being of the family (Government Decree, 2011). Vaccinations as part of health visits are a distinctive feature of the Finnish system (Nieminen et al., 2023). Ideally, families are seen by the same PHN over time, supporting continuity in care (Yokoyama et al., 2018). CHC services focus on the family as a unit of care (Poutiainen et al., 2014), requiring nurses to acknowledge the perspectives of both children and parents, while ensuring that the child's best interests are prioritized (Coyne, 2015; Mathisen Haaland & Bondas, 2024).

Children's participation in healthcare has increasingly been recognized as a core element of high-quality nursing. As such, children's participation supports their ability to make health-related decisions (Skauge et al., 2021), reduces treatment-related fears (Gilljam et al., 2016; Graber et al., 2024) and calms and empowers children (Wennström et al., 2011). It also improves the overall care experience (Graber et al., 2024; Nowak et al., 2020), and facilitates nursing practice (Banks et al., 2013). Good nursing care is based on respectful and open communication between the patient and the nurse (Meleis, 2018), and also on helping patients use their own abilities and take care of themselves (Bergbom et al., 2022). Participation can only occur within such a relational encounter (Antonovsky, 1987), and nursing care in the interaction among the nurse, the patient and the environment (Orem et al., 2003). Previous research highlights the importance of holistic nursing, diverse communication methods and the skilful use of caring practices in supporting children's involvement (Ortju et al., 2022).

Despite growing interest in patient participation, the perspective of young children have received limited attention in research. Young children, defined here as those aged between five and seven years, are often not included in healthcare research due to assumptions that they are not developed enough and depend too much on adults. However, from a holistic nursing perspective, children must be seen as individual and complete persons with their own views and experiences (Bergbom et al., 2022; De Vries & Rings, 2019). While some studies suggest that children wish to be more involved in their healthcare (Gilljam et al., 2016; Sjöberg et al., 2015), recent research focusing specifically on younger children remains limited. Although child participation has been widely studied in several settings, less is known about how young children themselves experience participation and care in child health clinic nursing encounters.

Ethical and legal frameworks including international human rights conventions, Finnish legislation and guidelines for nursing highlight the responsibility of nurses to support children's rights to participation, dignity and the expression of their views. These frameworks also highlight the nurse's professional responsibility to support these rights and to be mindful of the inherent power imbalance in encounters with children and families (ICN, 2012).

To address these knowledge gaps, this study employs a multidisciplinary, multi-method design to examine children's perspectives on nursing encounters at CHCs. The aim was to explore young children's perspectives on participation and care experiences in child health clinic nursing encounters.

The research questions were:

*RQ1*: What issues do children raise when talking about CHCs?

*RQ2*: What is relevant to children during a CHC visit according to their narratives?

## Materials and methods

### Design

This study used a qualitative multi-method design. The study can be divided into three phases. First, the data were collected with children using creative and participatory methods, and their interpretations were recorded. Secondly, the data were created with parents, and their interpretations of the children's data were recorded. Thirdly, a multi-method analysis was implemented with researcher triangulation, combining content analysis with semiotic analysis to support a more reliable interpretation of the children's narratives.

Although parents contributed contextual interpretations, the focus of the study remained on children's narratives. Adult perspectives were used only to support and contextualize the interpretation of children's data, not as separate findings.

### Theoretical framework

The following principles of the studies of children's perspectives provided the basis for study design: an emphasis was placed on interaction between the researcher and children, diverse communication methods were used, children's assent was confirmed, their needs and wishes influenced the research and the analysis emphasized their perspectives (Christensen & Prout, 2002; Karlsson, 2021). By combining presentational and representational data collection methods, the understudied research phenomena can be described from the views of people who have experience with it. The descriptive approach is flexible and allows viewing the phenomenon from different directions and without biases (Ortju et al., 2024).

Epistemologically, the study is situated within the framework of nursing science and interdisciplinary childhood studies. Theoretical foundations of family nursing (e.g., Friedman et al., 2003), as well as theories grounded in the concept of agency in nursing (e.g., King, 1981; Orem et al., 2003), have informed both the research design and the interpretation of the findings. These frameworks guided the researcher's choice of participatory and multi-method data creation, supporting the focus on children's and parents' interpretations as expressions of agency and shaped the analytic lens through which the family dynamics and children's perspectives are interpreted.

### Study setting, recruitment and inclusion criteria

The study was conducted in the region of North Karelia, Finland. Participants were recruited from one preschool group in the early childhood education and care (ECEC) centre in August 2022. Inclusion criteria for children were:

1) Age between 5 and 7 years.
2) Finnish as their native language. All parents of eligible children were invited to participate. Inclusion criteria for parents were being the guardian of a participating child.

No exclusion criteria were applied to children or parents based on the developmental level, communication abilities or other characteristics. Consequently, one child who did not use spoken language participated. Individualized communication aids, such as a picture folder and a visual communication device, were used to support the interaction with this child. In addition, all participating children were considered to benefit from the use of visual and object-based communication methods. Alternative communication methods were particularly employed when informing children about the study and seeking their assent to participate (see Ortju et al., 2024).

A research bulletin was distributed to parents and the personnel of the ECEC centre provided the signed permits for the researcher. Children were informed as a group using picture and object communication. Data were produced between October and December 2022. Written informed consent was obtained from the parents of all minor participants for both their own and their child's participation. In addition, assent was sought from the children using visual aids, object-based communication and gestures to ensure understanding and voluntary participation.

### Data collection

The first author conducted research-initiated role-play (RIRP) with three small groups of 3–4 children. Sessions were held in the local CHC. A daycare worker acted as a research assistant. Her role was to bring the children to the clinic with a researcher and hold one camera. Children were instructed to pretend a role-play, where at least one of them was a nurse and one was a child. The children were told that the play concerned a visit to the child health clinic. The researcher did not provide a detailed script or predetermined topics, to avoid directing the children's narratives. However, because young children may not clearly distinguish between different healthcare settings, references to other healthcare encounters were accepted as part of their broader healthcare experiences and were considered in the interpretation. Children were allowed to change roles during the play. Free movement and the usage of all equipment were allowed. Children had picture cards that could be used to interrupt the session.

The first author conducted the stimulated recall interviews (SRIs) in the ECEC centre. Each small group participated together with the same children who were together in the RIRP. Children were allowed to draw, paint and tell stories using the Storycrafting method (see Karlsson, 2013). Along with their creative activity, the researcher asked questions about their experiences in CHCs. The videotaped RIRP was presented, and children gave their interpretations of it.

Parents' SRIs were conducted in the ECEC centre by the first author. Children's videotaped RIRP and artwork were shown, and stories were read as a trigger for conversation. Parents were invited to share their interpretations of the data and opinions and experiences about CHCs. The researcher also asked questions about the family's healthcare experiences and how the adults and children interact during the health visits. The sessions were recorded on video. The first author made field notes immediately after each data collection session. In present study, parents' interpretations of data were considered in children's data analysis.

### Data integration and triangulation of analysis

The data comprised approximately 13.5 h of videotapes, 110 pages of transcriptions, 12 stories, 13 paintings and 15 drawings. The first author transcribed recordings verbatim after each session. Transcriptions were written in a Word document and all names were converted as pseudonyms. Transcribed SRIs included children's interpretations of their data. Artwork were described verbally, and stories were transcribed from field notes. All transcriptions were imported to ATLAS.ti 23.3.4 software.

The analysis proceeded in three distinct but connected phases. First, an inductive content analysis was conducted to identify the issues that children raised in their narratives, role-play, stories and artwork. This phase followed the qualitative content analysis process by Elo and Kyngäs (2008), including preparation, open coding, grouping, categorization and abstraction.

Second, a semiotic analysis was conducted as an interpretive complementary analysis to explore emotional, relational and symbolic meanings in selected multilayered data units. Third, the findings from the content and semiotic analyzes were integrated in a compiling analysis, in which the authors examined how the results addressed the research questions and the overall aim of the study. In the compiling analysis, the first author compared the categories generated through content analysis with the interpretive categories generated through semiotic analysis. The authors then discussed which findings appeared across several methods, which issues were emotionally or symbolically meaningful. The final compiled themes were agreed upon through co-author dialogue. Earlier research was considered when interpreting and discussing the findings, but it did not provide a coding frame for the inductive content analysis.

#### Inductive content analysis

The inductive content analysis followed the process described by Elo and Kyngäs (2008). The preparation phase included repeated reading of the data and selection of meaning units. In the organizing phase, meaning units were openly coded, grouped into subcategories and further abstracted into generic categories. The unit of analysis was a unit of meaning, which ranged from a few words to some sentences. A total of 425 citations were identified: 159 from RIRPs, 150 from SRIs, 68 from stories and 48 from artwork. Totally 72 codes, 19 subcategories and 5 generic categories were modified. Table I describes an example of analysis by one citation and its categorization from each method. The content analysis focused primarily on manifest content, while interpretive meanings were further explored in the semiotic analysis.

**Table I.** Example of inductive content analysis: one citation from each data collection method as an example for coding and categorization.

Method	Citation	Code	Subcategory	Generic category
Stimulated recall interview (SRI)	*Aino*: I was vaccinated. *Researcher*: How did it feel? *A*: Well, I screamed. *R*: Oh. *A*: I don't like vaccines. It's dumb.	Vaccinations are uncomfortable	Vaccinating	Nursing care activities
Researcher-initiated role play (RIRP)	Aino takes the plaster, puts it on Venla's hand and says calmly: Okay, I'll put it on. Hold still.	Putting on a plaster		
Painting	Painted image of a fairy and a vaccine syringe. Blood is coming out of the fairy and a plaster is painted next to it. The picture also shows flowers, butterflies and a rainbow.	Vaccination is an essential part of a CHC visit		
Drawing	Drawn image of the scale.	Measurement of weight	Physiological measurements	
Storycrafting	And then they told her that they would check her eyesight. So she said, “A, B, C, D, E, F, G”. She got everything right.	Eye examination		

After the inductive categorization had been completed, a descriptive supplementary examination was conducted by calculating the frequencies of subcategories and codes and by classifying selected expressions as positive, neutral or negative (example in Table II). This supplementary quantification was used only to support the interpretation of recurring issues and their emotional tone; it did not guide the formation of the categories. This step helped to make visible whether recurring issues were mainly described as pleasant, unpleasant or neutral by the children.

**Table II.** Example of classification citations as positive, neural of negative for an inductive content analysis.

Method	Citation	Subcategory	Classification
Stimulated recall interview (SRI)	*Researcher*: What do you think about when a vaccine is given? *Emma*: Uh. It stings. *R*: It stings a little, yeah. Is it a good thing or a bad thing that you get the vaccine? *E*: A good thing. *R*: Good thing, yep.	Vaccinating	Positive
Researcher-initiated role play (RIRP)	Anni takes a vaccine syringe and vaccinates Oliver. She tries to vaccinate Viola as well, but Viola hides under the table. *Viola*: No, no, Anni. No, no, no, no, no.		Negative
Storycrafting	My mummy and I went to the clinic and I was vaccinated there. And when the vaccination ended, we got out of there.		Neutral
Drawing	Drawn image of a child, her mother and a nurse in a CHC.	Presence of family members	Neutral
Painting	A painted picture of a nurse and a child in a CHC. The child's face painted fearful. Concerning the painting, the child says that being alone without her mother is scary and when her mother comes, she will be comforted.		Positive

#### Semiotic analysis as an interpretive analysis

To further analyze the data, a semiotic approach was conducted. According to Barthes (1964), semiotic analysis delves into various meanings expressed through signs and symbols and considers the intentions of the interactors and the context in which they communicate. The analysis assessed the atmosphere, non-verbal communication and the researcher's role. The contribution to semiotics by the structuralist linguist Jakobson (1960) in the form of six functions of communication was used. Within Jakobson's theory, the emotive function describes the relationship of the narrator to the issue being referred to, and the conative function shows the recipient's interpretation of the message. Between these interpretations are other analytic functions. The poetic function focuses on the message and how it is expressed, the referential function explains the context, the phatic functions shows the communication channel and the metalingual function explains the meaning and shared understanding between the speaker and the listener (Jakobson, 1960).

Three authors implemented analysis and consulted a method expert. The first author selected 54 data units: 6 from RIRPs, 25 from SRI's, 8 from stories and 15 from narratives about artwork. Units were selected if multilayered interpretations were found in the former analysis. The multidimensionality of the data required systematicity. Each unit was accompanied by a description of where, how and in whose presence the material was produced. After selection, the authors made analysis simultaneously: the first author with all 54 units and other two with five units each. The first author compared analyzes and proposed a conclusion, which was accepted by the others. In total, 116 conative functions were derived (example in Table III). To perceive the results, categories of conative functions were formed (example in Table IV). Participants' interpretations were considered in the analysis.

**Table III.** Example of semiotic analysis.

Citation from SRI		
*Researcher*: What do you think is a good nurse? *Linnea*: Well, I think such a nice nurse offers everything you ask for. *R*: Offers everything. What does she offer? *Linnea*: Well, if you get to play or draw. *Leevi*: Well, when I had my first therapist, so I got to decide for myself, for example, playing with myself. *R*: Yeah, it's nice to be able to decide yourself. *Leevi*: But now I got… A long, long time ago, so... *R*: Yeah. Now you've got someone else, right? *Leevi*: Yeah. *R*: Is that nice too? *Leevi*: Well, it is, it's a nice guy. *Researcher*: Okay.		
Functions of communication		
*Emotive function*: The children speak happily about the nurses. Leevi rolls his eyes while pondering what to say about his new therapist.	*Poetic function*: A conversation among the researcher and three children during the cleaning of painting equipment.	*Conative functions*: 1) Children describe healthcare clinics as nicer if play is allowed there. A nurse feels comfortable if she provides meaningful activities for the child and leaves time for free play and activities. 2) Children's past experiences influence their stance towards healthcare professionals. The children compare the adults who treated them.
	*Referential functions*: 1) Drawing and playing in CHC. 2) Playing in therapy. 3) Change of therapist.	
	*Phatic function*: The researcher asks the children a question, which two children answer. Leevi's father later says that it is important for Leevi to interact with a professional and if he feels that the nurse is not a “good guy”, he will not cooperate. The father also says that Leevi needs time to get acquainted and prefers to go to a familiar professional.	
	*Metalingual functions*: 1) A nice nurse lets children play and draw in CHC. 2) A nice professional lets the child decide about some functions and gives time for free play. 3) The child compares professionals and a stance toward them depends on previous experiences.	

**Table IV.** Example of categorization of conative functions in the second phase of the semiotic analysis.

Conative function	Subcategory	Generic category
Sometimes the child is annoyed by visiting a CHC and then receives a reward from her mother.	Rewarding relieves bad feelings.	Rewarding is important.
The children say that the reward after vaccination makes them feel better.		
(--) but feels the prize for it is important.	The prize is important.	
The child hopes to receive a reward for the clinic visit.		
Children report that they can receive prizes from the clinic, such as a lollipop or chewing gum.		
An award for a child from a clinic, such as a lollipop, is important.		
Children show in RIRP that the plaster is an essential part of vaccination. The children describe that the plasters are great.	The plaster is perceived as a reward.	
After vaccination, a plaster is applied.		

Parents' interpretations were used as contextual information in the semiotic analysis, particularly when interpreting children's non-verbal communication, references to previous experiences and meanings that were difficult to understand from the children's narratives alone.

### Ethical considerations

Legal guardians gave their written consent as required (TENK, 2019). For ethical reasons, children's assent to participate was taken using picture cards in every research situation, and their comfort and satisfaction were carefully observed during the process.

The study followed the principles of the Helsinki Declaration (WMA, 2013), which require the researchers to promote and safeguard participants' rights, health and well-being, and treat them with respect. As an underrepresented group in health research, young children were provided access to participate as the declaration states (WMA, 2013). Under the law in Finland (Data Protection Act, 2018) and the guidelines of the Finnish National Board on Research Integrity (TENK, 2019), this research did not require a statement from an ethical review board. The personal data of the participants were processed with special care, including storing the data as required by the EU General Data Protection Regulation (GDPR; EU, 2016/679).

The autonomy of the participants was respected when choosing the methods and places for data collection together. To respect equality, the results were sent to participants both in textual and in video format with simplified language. (Beauchamp & Childress, 2001, *p*. 88–93.) In practice, ethical considerations focused on supporting children's voluntary participation, monitoring their comfort during each session, allowing them to stop or change activities and adapting communication to each child's needs.

### Rigour and reflexivity

Rigour was ensured by conducting continuous critical discussions among authors. The methods were considered after every data collection. The input of the participants in interpreting the data strengthened the reflexivity of the study and aided in guaranteeing that the children's perspectives were clearly understood and explained in detail. A method expert confirmed that the analytical methods were used carefully and an understanding of the use of different methods was sufficient. While the researcher triangulation helped in developing shared understandings and revealing the deeper meanings in the data. Moreover, good research integrity was followed. The consolidated criteria for reporting qualitative research checklist (COREQ) was used while writing the manuscript (Tong et al., 2007).

The first author visited the ECEC group several times before data collection to build trust with the children. The Storycrafting method was rehearsed to ensure the children could use the method, and the children became familiar and comfortable with filming. The whole study was reflexive, and the researcher listened and observed children's behaviour, needs and wishes. Children could decide which methods they used, in which order and where. They could choose whether their actions were videotaped.

Selected analysis methods led us to interpret data in various aspects. Inductive content analysis was flexible and led us to analyze the data without strict theoretical frameworks. Theories and presuppositions were in the background, but the interpretation could look past them (Elo & Kyngäs, 2008). Quantification of qualitative data reduced the risks of bias and made it easier to detect recurring themes (Mikkonen & Kyngäs, 2020). Semiotic analysis helped us to notice the influence of background and environmental factors.

## Results

### Characteristics of participants

The study involved eleven children aged 5 to 7 years and twelve parents. Of the children, eight were girls and three were boys. Parents participated for ten of the eleven children: for two children, both the mother and the father participated; for eight children, only the mother participated; and for one child, neither parent participated. The parent participants therefore consisted of two fathers and ten mothers. The fathers participated together with the mothers as couples (two pairs), while the remaining eight mothers participated individually (eight one-on-one interviews). During the data generation sessions, the children were divided into three small groups of three to four participants.

Importantly, the participatory methods were not exclusive: the children were free to choose which activities they wished to engage in and could participate in as many or as few methods as they preferred. Ten children participated in role-play, and eleven took part in stimulated recall interviews. Six children told a story using the Storycrafting method, either individually or in pairs. Nine children drew pictures, and all eleven children engaged in painting.

### Results of content analysis

**Figure 1.**
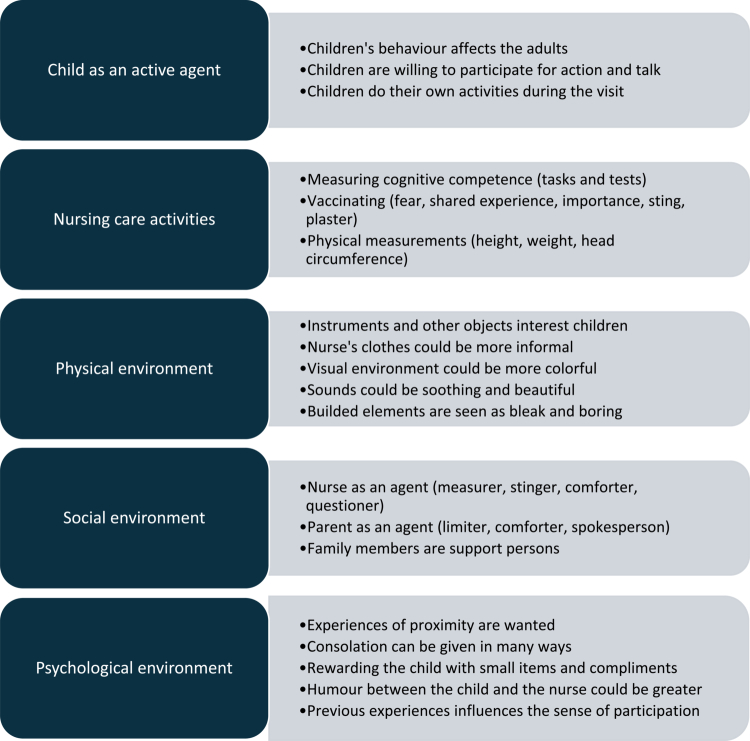
Results of the inductive content analysis: generic categories the children talked about.

The content analysis primarily addressed RQ1 by identifying the issues children raised when talking about CHCs. The children's narratives revealed a wide range of experiences and perceptions related to their CHC visits. Through inductive content analysis, these descriptions were organized into five interrelated generic categories that illustrate how children made sense of the encounters: (1) the child's active agency, (2) nursing care activities, (3) physical, (4) social and (5) psychological environment (Figure 1). Although some categories are named after care activities, they were derived from issues that children raised in their narratives, play, stories and artwork.

Regarding their agency, children described how their own emotions and behaviour influenced the course of the nursing encounters. When they experienced strong emotions like fear related to vaccinations or unfamiliar procedures, they might run away, refuse to cooperate, shout or hide. These reactions often led adults to set limits, give instructions or physically guide the child, which the children felt could make the encounter less positive.

Children also talked about responding to the questions and how they would like to move and touch the equipment. The children showed willingness to do own activities (e.g., playing) along with the visit. Regarding nursing care activities, they mentioned cognitive competence, physical measurements and vaccination. The children pointed out that instruments are interesting, and they would like to investigate them more. The visual environment, building elements and the PHN's clothes were described as dull. More colours and patterns were desired. The children dreamed of decorating the clinic themselves. They suggested that the CHC could have pleasant sounds, such as birds singing.

The children recognized the influence of adults on the course of their visits. They described parents sometimes restricting their activities and telling the PHN things about them. At the same time, both the PHNs and the parents were described as sources of comfort: children explained that adults provided consolation through soothing words, physical closeness and calm presence when they felt uncertain, afraid or experienced discomfort. Children most often named their parents, and in some cases a grandparent or sibling, as support person during the visit. The PHN was frequently described as the person who gives instructions.

Rewarding came up numerous times and appeared important, especially after the vaccination. Stickers, colourful plasters and other small rewards were seen as meaningful, and positive feedback from adults was also experienced as rewarding. The children talked about their previous health care experiences, which seemed to influence their attitudes towards nursing. Their narrations were often tinged with humour, which they hoped would also be present in CHCs.

**Figure 2.**
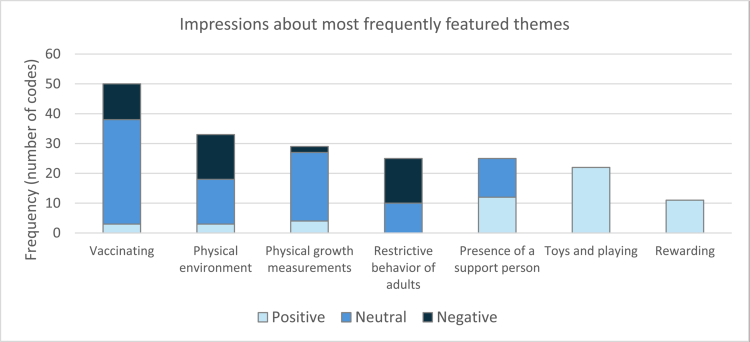
Results of the content analysis: children's impressions about the most frequently featured themes in children's narratives (f).

To comprehend the findings in a more profound way, the frequency with which different topics appeared in the data was examined. Most of the issues children associated with the CHC were only mentioned occasionally and were perceived as neutral or positive. Negative impressions were most often linked to vaccinations, aspects of the physical environment and restrictive behaviour by adults. Positive impressions were commonly connected with toys and play, rewards and the presence of a support person. The topics that appeared most frequently in the data were vaccinations, the physical environment, physical growth measurements, restrictive adult behaviour, support persons, toys and playing and rewarding (Figure 2). Figure 2 is included to support the answer to RQ1 by showing which issues appeared most frequently in children's narratives and how these issues were expressed as positive, neutral or negative experiences. The figure is not intended to quantify prevalence, but to make visible the recurrence and emotional tone of issues raised by the children.

### Results of semiotic analysis

The semiotic and compiling analyzes primarily addressed RQ2 by interpreting which issues appeared meaningful and relevant to children's participation and care experiences.

The semiotic analysis deepened these findings by highlighting the subtle meanings and emotional layers in the children's narratives. Seventeen interpretive categories were identified, reflecting how children experience and interpret different aspects of CHC visits. These categories were not intended to represent separate themes equal in weight, but to make visible the emotional, relational and symbolic meanings embedded in the children's narratives. They informed the compiling analysis by identifying which issues appeared especially meaningful for children beyond their literal verbal statements.

1) CHC visits can be frightening.
2) Some measures are unpleasant.
3) Adults determine the course of a visit.
4) Children feel they are being evaluated by adults.
5) Children want to be involved.
6) Play is important.
7) The presence and availability of support persons provide safety.
8) Rewarding is important.
9) Allowing enough time improves the care experience.
10) Moving around is natural for children.
11) Clinic's physical premises are important.
12) Children pay attention to the nature of the nurse.
13) Vaccination is an essential part of the visit.
14) Visits consist of various examinations.
15) Toys and care equipment are interesting.
16) Previous healthcare experiences affect children's perceptions.
17) Health visits are shared experience for the children.

### Compiling results

Together, these findings address the aim by showing how children described participation and care experiences through concrete activities, relationships, emotions and environmental features of CHC encounters. When integrating the findings from both analytic phases, several themes consistently appeared across all datasets and methods. Children spoke extensively about vaccinations across all methods and groups, describing both fear of vaccination and positive experiences of reward and the need for vaccination. Vaccination was not predetermined as the central topic of the role-play or interviews. Its prominence emerged from the children's own narratives across methods, suggesting that vaccination was one of the most memorable and emotionally salient aspects of CHC visits for them. Chidlren also highlighted growth measurements, sometimes even turning them into playful competitions, as meaningful elements of the visit.

**Figure 3.**
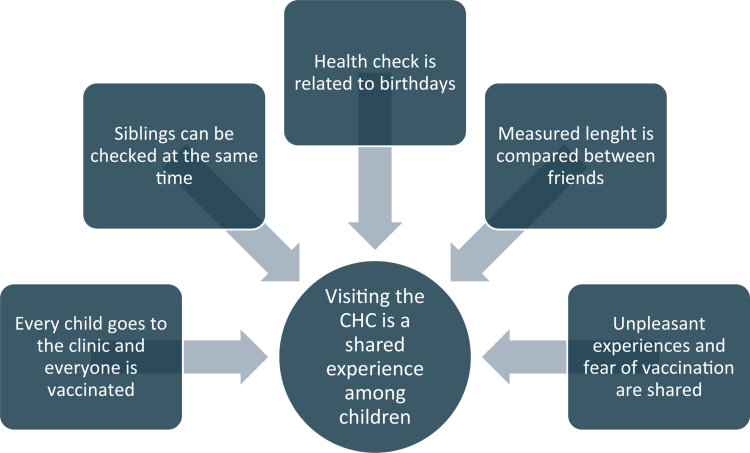
Results of the compiling analysis: children's experiences relating to CHC visits.

Children know that everyone go to the clinic and get vaccinated, and it is related to their birthdays. Children reported that sometimes health checks were carried out simultaneously with siblings. Visiting a CHC seemed to be a shared experience among children (Figure 3).

**Figure 4.**
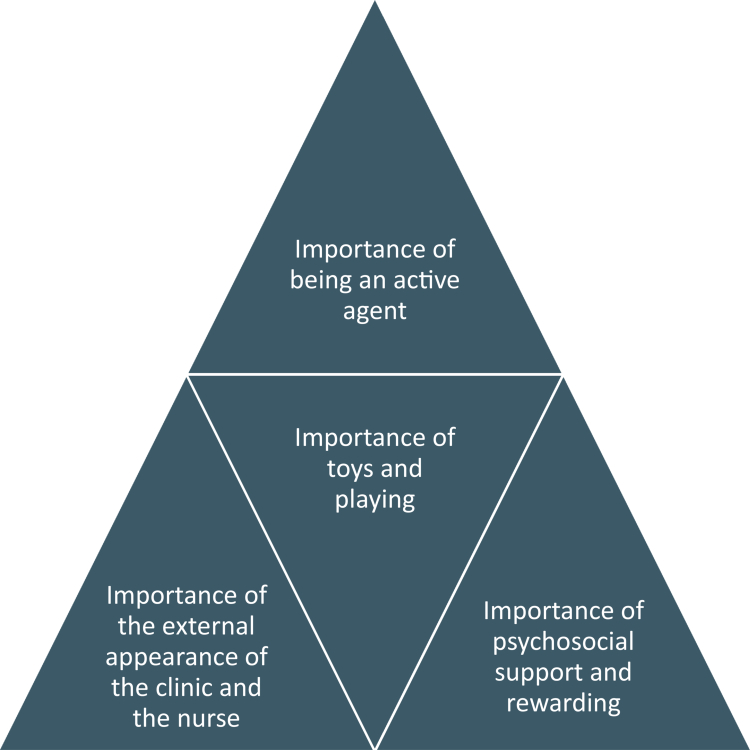
Results of the compiling analysis: issues relevant to children in terms of their inclusive care experience at the CHC.

The compiling analysis highlighted four recurring compiled themes that were interpreted as relevant to children in terms of their inclusive care experience (Figure 4).

The compiled analysis brought the children's perspective most clearly into focus by integrating what children talked about, how they expressed it and which issues appeared meaningful across methods. The four compiled themes below therefore represent issues that were especially relevant to children's inclusive care experience.

#### Importance of the external appearance of the clinic and the nurse

When asked in SRIs about what a pleasant CHC would be, the children's narration repeated desire for bright colours and beautiful pictures for the clinic premises: rainbows, hearts, flora, fauna and glitter. Children expressed hope about the colour and patterns of nurses' clothing. The parents' interpretation was that the children associated nurses' current uniforms with unpleasant experiences of being sick in a health centre. These descriptions suggest that the visual environment carries symbolic meaning for children and contributes to how safe or inviting the clinic feels.

#### Importance of playing

The children were repeatedly told that they were not allowed to use the toys available in the nurse's room. Some explained that toys are for predetermined tasks only. They described willingness to play and to look at toys and equipment. They said a CHC could have play areas or an entire visit could be arranged in a playhouse. Play and toys were seen as valuable in themselves and important as a means of comfort and reward. Toys clearly provided comfort during children's RIRP, where a child was afraid of the vaccination and became restless, and children acting as adults offered toys to calm her. The play calmed the child, and she assented to a vaccination. Overall, play not only emerged as an enjoyable activity but also as a coping resource and a way to regulate emotions during healthcare encounters.

#### Importance of psychosocial support and rewarding

The children's narration showed the need for psychosocial support. Staying alone with the nurse was considered with caution and the immediate availability of the parent was highlighted as important. The role of the support person was to be a giver of comfort and closeness. Experiences of proximity were also felt to be gained from the nurse. A relevant activity for all adults was rewarding children with praise or prizes, like stickers or patterned plasters.

#### Importance of being an active agent

The children described their agency through their narrations. In the RIRP, they described the unrest caused by the fear of vaccination and the expression of emotions through crying and screaming. Restrictive intervention by adults caused changes in their behaviour: children reported that when adults limited their movements, corrected their actions or imposed prohibitions, it made them feel uneasy and led to increased restlessness and reluctance toward nursing procedures. They also expressed that such restrictions felt unpleasant and reduced their willingness to cooperate. At the same time, the stories highlighted children's need to answer questions themselves and to be addressed directly, as well as their desire for self-motivated movement and the freedom to choose their position in the room.

Overall, the compiled findings suggest that children experienced participation not only as verbal involvement, but also through movement, play, proximity to trusted adults, sensory features of the environment and opportunities to influence small aspects of the encounter.

## Discussion

The central contribution of this study is that it foregrounds how young children themselves make sense of CHC encounters. From the children's perspective, participation was not limited to being asked questions or being allowed to answer. Rather, participation was experienced through opportunities to move, play, touch objects, remain close to a support person, receive explanations and be treated as the primary participant in the encounter. The research questions were used to operationalize the broader aim and guided the presentation of the findings.

The combination of nursing science and interdisciplinary childhood studies shaped the interpretation of the findings. From a nursing science perspective, the results highlight the importance of relational care, comfort, communication and the care environment in supporting participation. From the perspective of childhood studies, the findings emphasize children as active meaning-makers whose views are expressed not only verbally but also through play, movement, drawings, stories and embodied actions. Bringing these perspectives together helped to interpret participation as both a care practice and a child-centred experience.

This study described the factors that were relevant to children's participation and satisfaction in CHC visits. The children highlighted themes consistent with earlier research on young children in healthcare (Ortju et al., 2022) and older children in hospital settings (Davies et al., 2024), suggesting that children's fundamental needs for emotional safety, autonomy and meaningful interaction persist across contexts. A deeper understanding is provided by the findings through showing the ways in which the particular activities are interpreted by the children in CHCs, and what they remember as most important. These findings suggest that, for young children, participation is embodied and relational rather than only verbal. Children's sense of being included was shaped by how the encounter allowed them to act, move, play, seek comfort and interpret the environment around them.

Children's attention to vaccination, physical measurements and cognitive assessments underscores that the core activities of CHCs (Government Decree, 2011) are not merely routine procedures but central experiences shaping how children perceive the visit and evaluate its fairness, predictability and emotional tone. This aligns with an earlier study indicating that nurses' competence in selecting and tailoring activities is crucial for supporting children's participation (Ortju et al., 2022). In line with family nursing theory, the findings further highlight that shaping parents' attitudes and actions toward their child is important (Lind et al., 2024; Tanninen et al., 2016; Wong et al., 2023), as supporting the parents can improve the child's participation and protect the child's best interests are upheld. Overall, the results show that understanding the basic nursing principles, valuing the child and applying the art of caring are essential in public health nursing.

The children's narratives conveyed the wish that the caring environment could be more colourful, with things for the children to watch and touch. In Bishop (2012) study, children reported that artwork have positive effects on their comfort in the hospital. The artwork made by other children especially conveys the message that children are welcome (Bishop, 2012). Graber et al. (2024) and Davies et al. (2024) have proved that playing in hospitals is important to make children feel safe and welcome as children, not just patients. In this study, children expressed a desire to decorate the CHC facilities. Unlike children's hospitals in specialized healthcare (Graber et al., 2024), child-oriented planning has rarely been implemented in primary care.

The nurses' competence and skills in using communication methods have previously been found to be meaningful (Lind et al., 2024). In this study, children's views were understood through creative methods. This supports earlier research by Handberg and Voss (2018) showing that using different ways of communication methods helps the nurses to acquire important information about the needs of the patients that might otherwise be missed. Seen in a broader context, the findings indicate that child participation in primary healthcare depends not only on professionals' communication skills but also on everyday material, emotional and relational conditions that make participation possible for children.

In the children's narratives, different healthcare professionals were often grouped together, and they often talked about the nurses as doctors. Previous experiences related to the care of the children and their relatives became part of the narratives. The children's views described here can be considered a description of their relationship with healthcare professionals, not just with PHNs.

According to the results, children often perceive the restrictive behaviour of adults as neutral. In their narratives, parents or nurses limited children's movement, voice and touching of equipment during a visit. The children did not question whether the adults' actions were right or wrong, but instead seemed to accept them as normal. In the study by Davies et al. (2024), which was conducted in the context of specialized healthcare, children and adolescents described adult decision-making authority as dominant, often overriding the views of young people. The participants reported feeling unable to influence matters concerning themselves in the ways they would have preferred (Davies et al., 2024).

This study demonstrated the usability of the creative methods in nursing science. The knowledge generated on methods of producing data from child's perspectives can be used broadly in health sciences. The methods used can be applied to practical nursing care to bring information about children's views.

Taken together, these findings highlight why this research is important for primary healthcare practice. By showing how children themselves interpret CHC visits, the study brings forward aspects of the encounter that adults often overlook, such as the emotional meaning of the environment, the value of play, the importance of direct communication and the impact of adults' restrictive behaviours. These insights indicate that even small adjustments, such as allowing children more agency, ensuring access to play or providing clearer and more child‑friendly explanations, may significantly improve children's sense of safety, participation and trust. Enhancing these elements in practice can strengthen the quality of the visit, increase children's cooperation in procedures, reduce anxiety and support more positive long‑term attitudes toward healthcare.

### Strengths and limitations of the work

The trustworthiness of the study was supported by prolonged engagement with the children, the use of multiple data generation methods, researcher triangulation, participant interpretations and reflexive discussion among the authors. Credibility was strengthened by comparing findings across role-play, interviews, stories, drawings, paintings, field notes and parents' contextual interpretations. Transferability is limited by the context-specific sample, but the detailed description of the setting, participants and methods may help readers assess the relevance of the findings to similar CHC and primary healthcare contexts.

The primary strength was the usage of several methods of producing data and two methods of analysis, both of which brought new perspectives. Important strengths were reflexivity and sensitivity to children's needs and desires. Children were listened to and observed, and they were given enough time to build trust and express themselves. The ways of communication were broad, and children could choose how they communicated. According to Kara (2020, *p*. 29), allowing room for creative expression and open conversation might have elicited more interesting data than a structured process could have. In essence, the initiative aimed to foster a secure and enabling environment for children, thereby facilitating the expression of their views and experiences (cf. Davies et al., 2024).

The creative and participatory methods contributed to the study by enabling children to express experiences that might not have emerged through verbal interviews alone. Role-play, drawing, painting and Storycrafting allowed children to communicate through action, imagination, humour, bodily expression and visual symbols. At the same time, these methods also required careful interpretation, as children's expressions may be shaped by the research situation, group dynamics and the researcher's prompts. For this reason, the findings were interpreted across methods and with attention to participant interpretations and researcher reflexivity.

The three-phase analysis enabled both descriptive and interpretive understanding of the data. Content analysis identified issues raised by children, semiotic analysis added insight into emotional, relational and symbolic meanings and the compiling analysis integrated these findings. However, this approach also increased interpretive complexity and required careful reflexivity to avoid overinterpretation.

Healthcare studies often exclude those children who have disabilities (Njelesani et al., 2022). No exclusion criteria were applied based on disability or health condition. One child with communication challenges could participate by using personal aids, providing valuable information for the study. The exclusion based on disability would have impoverished the data and caused a serious ethical problem by possibly causing the child to feel abandonment and exclusion. Some exclusion criteria in healthcare research are unnecessary due to a lack of ability to include and understand diverse individuals. This means that respect for human dignity, equality and the understanding of individuality are few core strengths of this research. The participation of one child with communication challenges demonstrated the feasibility of inclusive data generation methods in this study context. However, no broader conclusions can be drawn from a single participant, and this finding should be interpreted cautiously.

However, the difficulty of interpreting children's communication can be seen as a limitation. Children are used to answering both verbal (Ponizovsky-Bergelson et al., 2019) and written tasks such as drawing (Backett-Milburn & McKie, 1999) as they think adults want. Therefore, the children were informed that there were no right or wrong answers, and that all ideas were important. Kara (2020, *p*. 158) explains that combining different types of data aids in illuminating diverse dimensions of the participants' experiences. No analysis method alone could have described such multidimensional data. Each stage of the process raised new questions, which guided the final stage of the analysis. Moreover, by asking for the participants' interpretations, the risk of misinterpretations was reduced. The reliability and usability of the data collection methods were evaluated, and the use of a genuine care environment in research, the recruitment of children directly from the ECEC centre, and the effects of the physical environment and group on children's narration were discussed (Ortju et al., 2024).

A limitation of the study is that the participants were recruited from one preschool group in one geographical area. This may have shaped the children's shared experiences and the topics that emerged in the data. Therefore, the findings should not be understood as statistically generalizable, but as context-bound insights that may be transferable to similar CHC and primary healthcare contexts. Another limitation is the time elapsed between data collection and publication. Although the central activities of CHC visits have remained relatively stable, societal and service-related changes may influence children's current experiences. This temporal gap was considered when interpreting the relevance and transferability of the findings.

The study's findings were based on a particular group of people, in a particular setting, at a certain time, which is typical in qualitative research. Especially in children's ways of acting and the narratives they tell, the environment and the lived experiences are affected strongly. We also acknowledge that the researcher's prompts and interpretations during the interviews may have influenced how children expressed their views. This was considered in the analysis by comparing interview data with role-play, stories, artwork, field notes and participant interpretations.

### Recommendations for further research

Based on the present findings, further research should examine how the issues children identified as meaningful—such as play, support persons, physical environments, opportunities for agency and emotionally salient procedures such as vaccinations—are addressed in everyday CHC practice. For example, further studies should explore how the issues raised by children are reflected in the everyday practice of PHNs. Observation of health visits could bring more understanding of the effects of the interaction. Secondly, various caring environments should be studied and tested. Thirdly, children should be included as active participants in studies that develop nursing and caring environments. Moreover, the PHNs' and parents' views on children's participation should be studied and compared with the children's views.

Apart from that, studying the impact of implementing the health visit partially or fully in the child's everyday environments at home or in the ECEC centre is also appropriate. The possibilities of digitalization should be studied from the aspect of the advantages and disadvantages of remote health visits and how the child could be better prepared. While, the feedback systems should be studied and developed from children's perspectives. Creative methods could help to determine children's views in nursing encounters. This should be clarified through more detailed studies.

### Implications for policy and practice

For nursing practice and the development of the care environments, the findings of this study have important implications. The implications for practice arise directly from the children's descriptions of what shaped their care experiences: opportunities to play and move, access to support persons, a welcoming physical environment, sensitive communication and the possibility to act as active participants during CHC visits.

First of all, the children should be actively involved in the planning and design of the healthcare environments. This is crucial because the factors that are relevant from the child's perspectives may be things that adults do not think about.

The results can be used by individual nurses, healthcare teams and organizations to improve practice and reflect on current approaches to care. For example, it is important to consider whether children are truly allowed to engage with available resources, such as toys or if these are only displayed without being accessible. Questions should also be raised about whether children are unnecessarily separated from their support persons, whether opportunities for free play are provided, and whether there is space for the children to express their own choices and initiatives. Additionally, healthcare professionals should reflect on whether they allocate sufficient time to listen to the children and respond to their needs, as this is essential for promoting meaningful participation and positive care experiences.

## Conclusion

In nursing, the Ottawa Declaration (WHO, 1986) highlights that promoting health involves strengthening individuals' agency and personal resources. This study demonstrates that, from young children's perspectives, participation and sense of inclusion in CHC visits were shaped by respectful interaction, opportunities for play and self-directed action, proximity to support persons and attention to the physical and psychosocial environment.

The children expressed their views not only verbally, but also through role-play, stories, drawings, paintings, movement, humour and emotional responses. Rather than requiring specialized expertise, supporting children's participation calls for professional sensitivity, the ability to recognize children's diverse ways of communicating and a genuine attitude that values the child as an active participant in the encounter. These qualities are essential in creating inclusive care environments where children feel seen, heard, safe and respected. Likewise, creative and participatory research methods proved to be valuable tools for capturing children's perspectives in nursing science (see also Ortju et al., 2024).

The findings highlight that children's experiences are shaped not only by clinical procedures but also by the physical and psychosocial environment, the presence of support persons and opportunities for play and agency. These insights are highly relevant for developing practices in primary healthcare and family nursing. The family healthcare services should be designed with children's perspectives as a central focus. Improving the child's care experience ultimately depends on the individual professional's commitment to listening, adapting practices and creating opportunities for children to express their views and participate meaningfully in their own care.

## Data Availability

The data that support the findings of this study are available on request from the corresponding author, XX. The data are not publicly available due to their containing information that could compromise the privacy of research participants.

## Clinical resources

Video about the Storycrafting method: https://youtu.be/X-8icqVIq18?feature=shared.

Semiotic analysis simply described: http://www.signosemio.com/jakobson/functions-of-language.asp.

Briefly about the Finnish CHC: https://stm.fi/en/maternity-and-child-health-clinics.
